# Persistence of hepatitis E virus in the liver of non-viremic naturally infected wild boar

**DOI:** 10.1371/journal.pone.0186858

**Published:** 2017-11-08

**Authors:** María A. Risalde, Antonio Rivero-Juárez, Fernando Romero-Palomo, Mario Frías, Pedro López-López, David Cano-Terriza, Ignacio García-Bocanegra, Saúl Jiménez-Ruíz, Ángela Camacho, Isabel Machuca, José C. Gomez-Villamandos, Antonio Rivero

**Affiliations:** 1 Unidad de Enfermedades Infecciosas, Hospital Universitario Reina Sofía de Córdoba, Instituto Maimonides de Investigación Biomédica de Córdoba (IMIBIC), Córdoba, Spain; 2 Dpto. de Anatomía y Anatomía Patológica Veterinaria, Facultad de Veterinaria, Universidad de Córdoba (UCO)—Agrifood Excellence International Campus (ceiA3), Córdoba, Spain; 3 Dpto. de Sanidad Animal, Facultad de Veterinaria, UCO—ceiA3, Córdoba, Spain; CEA, FRANCE

## Abstract

Hepatitis E virus (HEV) is an emerging zoonotic pathogen with pigs and wild boar serving as reservoirs for human infection through direct contact with infected animals or the consumption of raw or undercooked pork products. The liver is considered the main target site of HEV replication in swine and an important organ in the pathogenesis of the disease. The aim of this study was to characterize the target liver cells for HEV entry in naturally infected wild boar and to evaluate the type and severity of the pathological changes in order to reach a better understanding of the hepatic pathogenic mechanisms involved in hepatitis E. In total, 58 livers from hunted wild boar were histopathologically evaluated. The presence of specific HEV antibodies in serum was determined by indirect ELISA. Immunohistochemistry was used for the detection of HEV antigen and Real time RT-PCR to detect HEV RNA in liver and serum. HEV seroprevalence in these animals was of 5.197% (CI_95%_: 1.77–14.14). By Real time RT-PCR, HEV was detected in the liver tissue of four wild boar (6.8%; CI_95%_: 2.7–16.4) and only one animal was also positive in serum (1.7%; CI_95%_: 0.3–9.1). The non-viremic animals naturally infected with HEV presented evidence of liver infection, mainly in Kupffer cells and liver sinusoidal endothelial cells, without apparent associated hepatitis lesions. This study supports the hypothesis that low viral titers may persist in the liver of non-viremic individuals, giving thus the possibility of consumption of contaminated liver of animals diagnosed as HEV-negative in serum. Further immunopathogenic studies are necessary to elucidate the mechanisms responsible for this process and to evaluate the protocols of HEV diagnosis in animals destined for human consumption.

## Introduction

Hepatitis E virus (HEV) is an important human pathogen and the etiological agent of hepatitis E, an infection considered endemic in many countries of Africa and Asia [1, 2]. This virus has severe effects, such as fulminating hepatitis, and increases mortality by up to 20% in pregnant women [3], patients with chronic liver disease and immunosuppressed individuals (e.g. HIV-infected and transplant patients) [4]. Hepatitis E is also a zoonotic infection, in which pigs and wild boar (*Sus scrofa*) serve as reservoir species for HEV transmission to humans [5–8]. Widespread evidence indicates that endemic cases in industrialized countries are typically associated with direct contact with infected animals or the consumption of raw or undercooked pork or wild boar products [9–14].

In Spain and many other European countries, wild boar populations have notably increased [15]. This large game species is widely consumed by humans and is used for the commercial production of meat or in local products that are usually consumed without cooking [9, 14, 16], thereby increasing the risk of zoonotic transmission. As a result, the ubiquity of HEV infection in wild boar raises concern about the presence of the virus in hunted animals and the current lack of official control programs of this zoonotic agent.

The main target organ for HEV replication in swine is considered to be the liver [17–20]. HEV infection in pigs is usually completely unapparent [21, 22], although mild hepatic lesions characteristic of hepatitis have been described in experimentally [23] and naturally infected pigs [24]. Nonetheless, although the liver is considered an important organ in the pathogenesis of HEV, some aspects of the process remain unclear, such as the mechanisms of liver damage or the role of the liver in viral reactivation. The purpose of this study was to characterize the hepatic target cells of HEV in naturally-infected wild boar and to evaluate the type and severity of the pathological changes produced, in order to gain a better understanding of the hepatic pathogenic mechanisms involved in hepatitis E.

## Materials and methods

### Study population

A total of 58 wild boar were randomly sampled during the hunting season in 5 hunting areas in Andalusia, southern Spain (36°N–38°600 N, 1°750 W–7°250 W). All hunter-harvested wild boar samples were collected between October 15 in 2015 and February 15 in 2016. Individual data on gender and age were recorded for each animal. Age was determined on the basis of tooth eruption and animals that were under 12 months old were classified as juveniles, those between 12 and 24 months as sub adults, and those over 2 years old as adults.

At postmortem examination, a whole blood sample was taken from all the animals using puncture of the cavernous sinus of the dura mater to obtain the serum [25]. Liver samples from all hunted wild boar were fixed in 10% buffered formalin solution for histopathologic study; a piece of each liver was also submerged in RNA*later*® Stabilization Solution (Thermo Fisher Scientific Inc., Waltham, MA, USA) and frozen at—80°C for subsequent Real time polymerase chain reaction (RT-PCR) analysis. The time period between the death of the animals and the sampling was of 2–4 hours and the samples were immediately processed for molecular and histological methods as was described in other studies [5, 15].

### Ethics statement

This study did not involve purposeful killing of animals. Professional personnel collected blood and liver samples mostly from hunted-harvested wild boar during the hunting season. These animals were legally hunted under Spanish and EU legislation and all the hunters had hunting licenses. No ethical approval was deemed necessary; the collection of all the samples was being performed for routine procedures before the design of this study in compliance with the Ethical Principles in Animal Research. Thus, blood or liver samples were not collected specifically for this study. Protocols, amendments and other resources were done according to the guidelines approved by each Autonomous government following the R.D.1201/2005 of the Ministry of Presidency of Spain.

### Molecular study

Viral RNA was extracted from 200μl of serum with the commercial QIAmp MinElute Virus Spin Kit, and from the liver with the RNeasy Mini Kit (QIAgen, Hilden, Germany), using automated procedures (QIAcube. QIAgen, Hilden, Germany). Samples were frozen at -80°C until analysis. For the diagnosis of HEV infection, Real time RT-PCR was performed using the LightCycler 480 (Roche. Basel, Switzerland), as described by Abravanel et al. [26]. For the reaction, the QIAgen One step PCR Kit (QIAgen, Hilden, Germany) was used. The primers (15μMol) used were sense HEV5260 (5´-GGTGGTTTCTGGGGTGAC-3´) and antisense HEV5330 (5´-AGGGGTTGGTTGGATGAA-3´). The probe employed (20μMol) was the HEV5283 (5´-FAM-TGATTCTCAGCCCTTCGC-TAMRA-3´). The thermal profile was 50°C for 30 min and 95°C for 15 min, followed by 45 cycles of 94°C for 10s, 55°C for 20s and 72°C for 60s. An external in run standard curve was applied to calculate HEV viral load using the WHO HEV standard strain (Gen Bank: M73218) supplied by the Paul-Ehrlich-Institute (code 6329/10).

### Serological study

The presence or absence of HEV antibodies (Ab) in serum was confirmed by a commercial indirect enzyme-linked immunosorbent assay (ELISA) (PrioCHECK™ HEV Antibody ELISA Kit, porcine. Thermo Fisher Scientific, Massachusetts, USA), following the manufacturer’s instructions.

### Pathological examination

The formalin-fixed samples were dehydrated through a graded series of alcohol to xylol and embedded in paraffin wax. After staining with hematoxylin and eosin (H&E), liver sections of approximately 1 cm^2^ were digitally scanned with a NanoZoomer 2.0-HT scanner (Hamamatsu Photonics, Japan) and two experienced observers performed a blind semiquantitative histopathologic evaluation. For the analysis, NDP.view2 viewing software (Hamamatsu Photonics) was used to accurately quantify the number and size of mononuclear cell infiltrates. A detailed explanation of the scoring system is provided in the footnotes of Table 1.

**Table 1:** Histopathological evaluation of liver tissue and results of IHC, serology and real-time PCR in HEV-positive wild boar.

Animal	Sex	Age	Real-time PCR HEV		Liver HEV viral load (copies/mL)	IHC HEV	Ab HEV	Hepatocellular necrosis	Lymphoplasmacytic aggregates	Sinusoidal congestion	Bile pigment accumulation
			Liver	Serum							
1	Female	Subadult	+	-	532	+	-	/	♦	▲	/
2	Female	Subadult	+	-	478	+	-	⸹	♦	▲	●
3	Female	Juvenile	+	-	107	+	-	/	♦	▲▲	/
4	Female	Adult	+	+	426	+	-	/	♦♦	▲▲▲	●

Real-time PCR hepatitis E virus (HEV), immunohistochemistry (IHC) HEV and antibodies (Ab) HEV: (-) negative; (+) positive.

Hepatocellular necrosis:(/) absence; (⸹) <10 cells per total section of 1cm^2^ approx.

Lymphoplasmacytic aggregates: (/) absence; (♦) ≤3 small aggregates; (♦♦) 4–7 small or medium-sized aggregates.

Sinusoidal congestion: (▲) very scarce presence of erythrocytes; (▲▲) ≈30% of sinusoids contain moderate amounts of erythrocytes; (▲▲▲) 30–80% of lobules are highly congested.

Bile pigment accumulation (extracellular (canalicular cholestasis) and intracellular): (/) absence; (●) multifocal and in low amounts.

Sections of the formalin-fixed paraffin-embedded liver tissue samples were routinely processed for immunohistochemistry (IHC) using the avidin-biotin-peroxidase complex (ABC) method described by Schlosser et al. [19] with some modifications. Briefly, endogenous peroxidase activity was exhausted by incubation with 0.3% hydrogen peroxide in methanol for 30 minutes at room temperature (approx. 25°C). The sections were incubated with 0.2% proteinase K (Sigma-Aldrich, San Luis, Misuri, USA) in 0.05 M Tris buffered saline (TBS; pH 7.6) for 8 minutes at 37°C in oven for antigen retrieval; after pretreatment, sections were covered with 20% normal goat serum (Vector Laboratories, Burlingame, CA) in 0.01M phosphate-buffered saline (PBS) at room temperature for 30 minutes. After blocking, the sections were incubated with a rabbit anti-HEV gt3 hyperimmune serum (rHEVgt3-HIS) in a 1:500 dilution at 4°C overnight (approx. 18 hours). The sections were then incubated for 30 minutes at room temperature with biotinylated goat anti–rabbit IgG secondary Ab (Vector Laboratories, Burlingame, CA) diluted 1:200 in TBS containing 10% normal goat serum. All tissue sections were finally treated with ABC complex (Vectastain ABC Elite Kit; Vector Laboratories) for 1 hour at room temperature, then rinsed in TBS and incubated with the chromogen solution (NovaRED Substrate Kit; Vector Laboratories). Finally, slides were counterstained with Harris’s hematoxylin. Tissues from wild boar without HEV infection in liver or serum, confirmed by Real time RT-PCR, were used as negative controls. As positive control was used a liver sample from a pig experimentally infected with HEV and with liver infection confirmed by Real time RT-PCR and IHC.

Identification of the different immunolabeled cell types was based on morphological features, location and size of the cells.

### Statistical analysis

HEV prevalence was estimated from the ratio of positive samples to the total number of samples analyzed, with exact binomial confidence intervals of 95% (CI_95%_). Prevalence by age and sex was also evaluated. Frequencies were compared using the χ2 test or Fisher’s exact test, and significance was set at a two tailed p-value of less than 0.05. A bivariate analysis was carried out to discover the variables related to HEV infection. Analyses were carried out using the SPSS statistical software package, version 18.0 (IBM Corporation, Somers, NY, USA) and Winpepi software, version 11.36 (Brixton Health).

## Results

Of the total of 58 wild boar included in the study, 22 were males and 36 females; 6 wild boar were classified as juveniles, 15 as sub adults and 37 as adults (Table 1). HEV was consistently detected by Real time RT-PCR in the liver tissue of 4 of the wild boar examined (6.8%; CI_95%_: 2.7–16.4) and only one of these was also positive in serum (1.7%; CI_95%_: 0.3–9.1). The rate of HEV infection was higher in females than males (11.1% vs. 0%, p = 0.22; OR = 6.23, CI_95%_: 0.31–121.55). Prevalence rates according to animal age were 20% in juveniles, 15.3% in subadults and 2.7% in adults; no significant differences in prevalence rates were found between adults and non-adults (p = 0.3; OR = 5.48, CI_95%_: 0.21–140.91). Viral RNA was not detected in the serum of wild boar with HEV-negative livers.

Antibodies against HEV were detected in 3 wild boar out of 58 animals analyzed (5.19%; CI_95%_: 1.77–14.14), 2 of them were adults (seroprevalence in adults was 5.41%; CI_95%_: 1.5–17.7) and 1 juvenile (seroprevalence in juveniles was 16.6%; CI_95%_: 3–56.3). None of the animals HEV-positive in liver or serum presented antibodies against the virus.

The histopathologic evaluation of the livers of wild boar naturally infected with HEV did not present any remarkable pathological change specific of viral hepatitis compared with the HEV-negative wild boar. In general, the presence of mononuclear infiltrates was the most common lesion observed in the livers of the hunted wild boar infected with HEV.

An overview of the histopathologic lesions as well as IHC, serology and Real-time PCR results of the HEV-positive wild boar is given in Table 1. These data in HEV-negative wild boar are represented in S1 Table.

**Fig 1:**
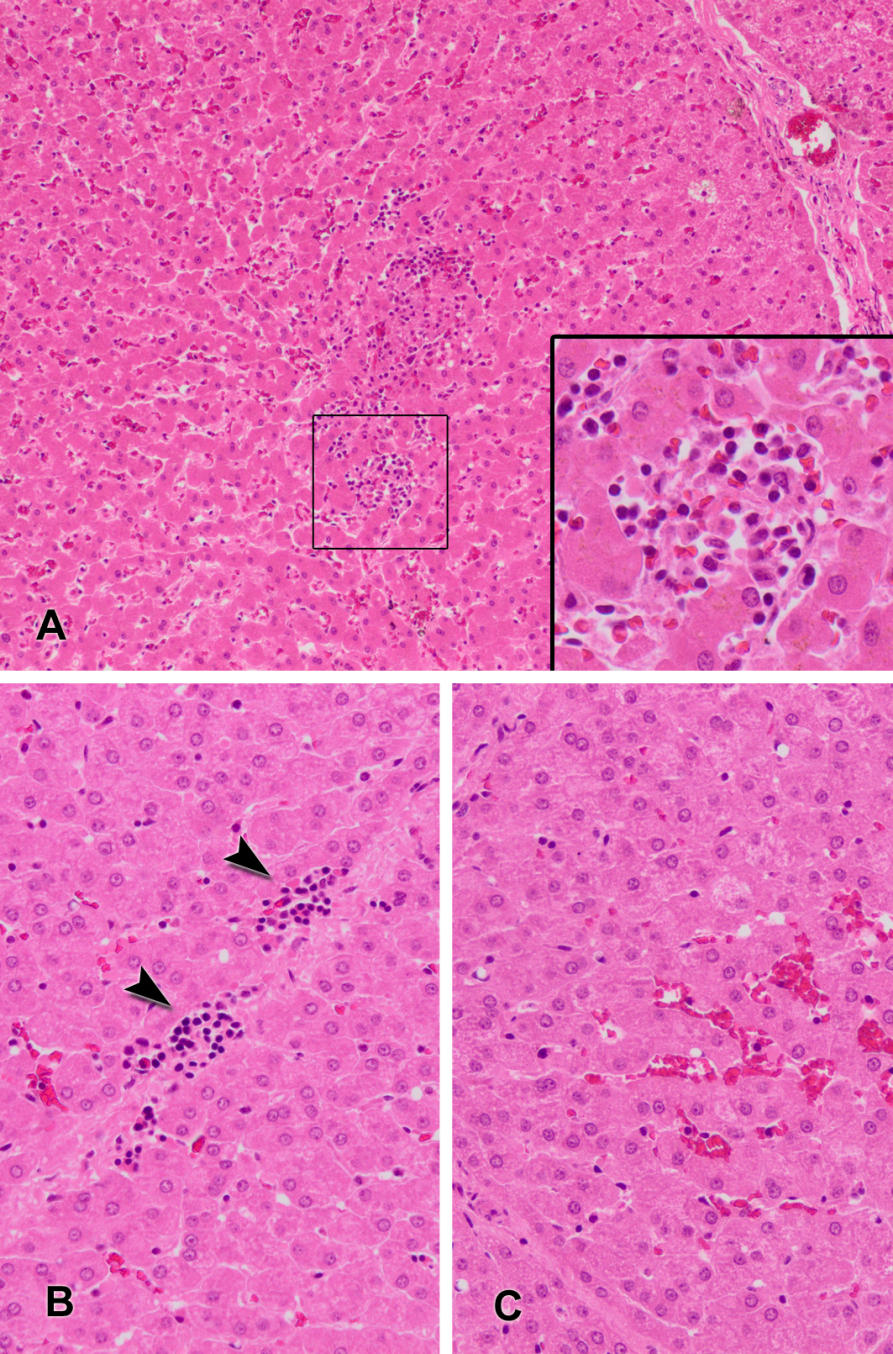
Histopathologic lesions in wild boar naturally infected with hepatitis E virus. Fig 1A: liver with presence of mild intralobular lymphoplasmacytic aggregates in the hepatic parenchyma. Inset showing a lymphoplasmacytic aggregate at higher magnification (hematoxylin and eosin, HE). Fig 1B: liver showing slight perilobular infiltrations of lymphocytes (arrowheads) (HE). Fig 1C: hepatic lobule with moderate hyperemia of liver sinusoids (HE).

The livers of the 4 animals with naturally occurring HEV infection displayed only mild diffuse lymphoplasmacytic infiltrates (Fig 1A and 1B), with one of them also showing slight signs of hepatocyte necrosis. One of these animals also had a high degree of congestion in the hepatic sinusoids (Fig 1C).

**Fig 2:**
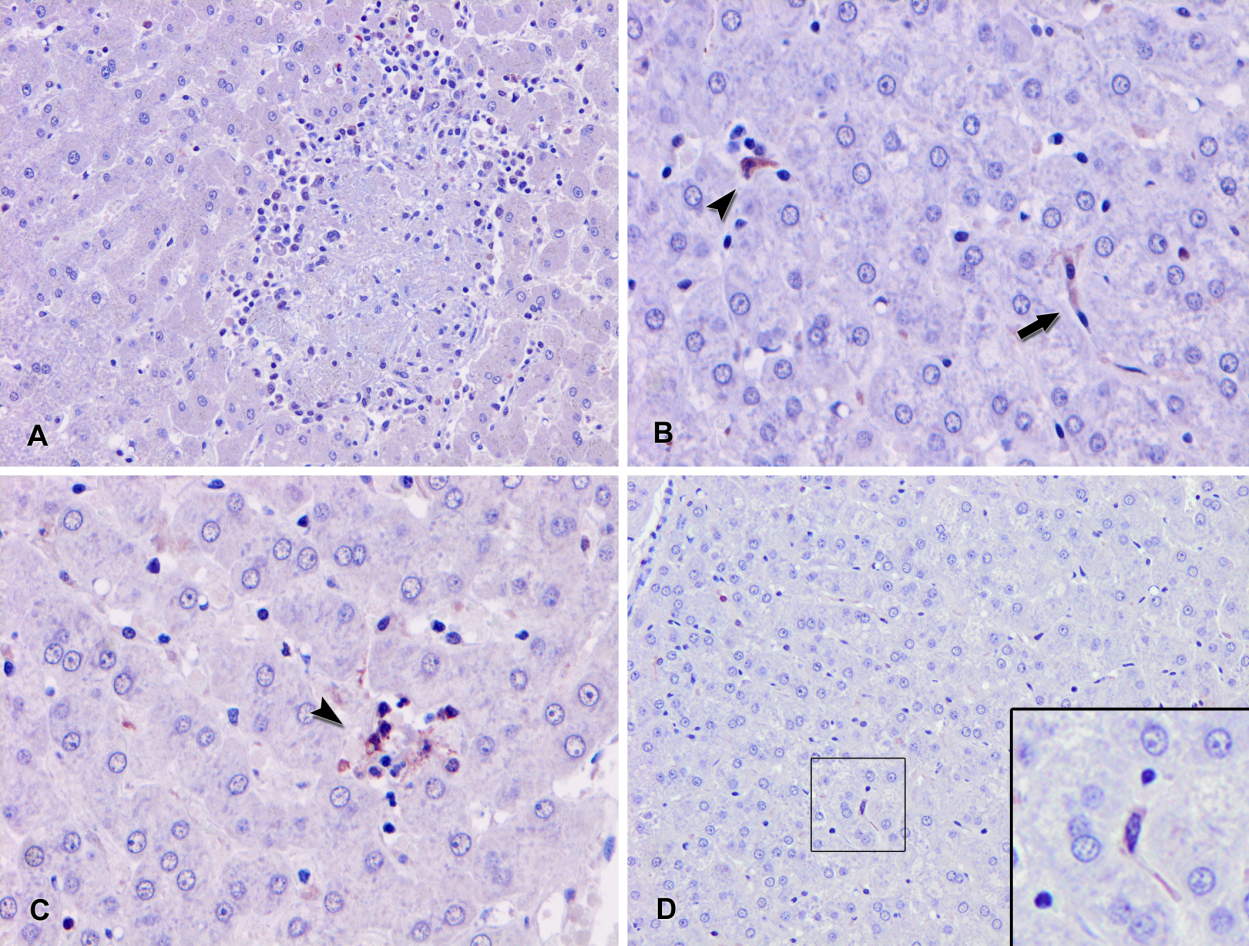
Representative photomicrographs of liver sections from 3 wild boar naturally infected with hepatitis E virus. Fig 2A: liver with absence of association of the HEV antigen inside the lymphoplasmacytic aggregates (Immunohistochemistry (IHC) with the avidin-biotin-peroxidase complex (ABC) method counterstained with hematoxylin). Fig 2B: hepatitis E virus (HEV) immunolabeling in the cytoplasm of the Kupffer cells (arrowhead) and the sinusoidal endothelial cells (arrow) (IHC with ABC method). Fig 2C: HEV immunostaining of a light lymphoplasmacytic infiltrate (arrowhead) (IHC with ABC method). Fig 2D: liver showing slight labeling for HEV antigen in the hepatic parenchyma, which is appreciable in the Kupffer cells (inset) (IHC with ABC method).

IHC using a rabbit anti-HEV gt3 hyperimmune serum (rHEVgt3-HIS) located viral antigens in the hepatic tissue of the 4 animals with HEV detected by Real time RT-PCR, while no immunohistochemical signals were observed in the liver of the other wild boar in which HEV RNA was not detected. HEV antigen in hepatic tissue was found mainly in the Kupffer cells and liver sinusoidal endothelial cells, in multifocal lobules not associated to hepatic lesions (Fig 2). Small numbers of hepatocytes and some interstitial macrophages were positive for HEV antigen. In the hepatic cells, HEV-specific labeling appeared as evenly distributed dark red or diffuse granular patterns of cytoplasmic staining.

## Discussion

The present study assessed the lesions and distribution of HEV in livers from hunted wild boar. Three non-viremic animals with naturally occurring HEV infection presented evidence of viral replication in liver, mainly in the Kupffer and sinusoidal endothelial cells, although not associated with characteristic hepatitis lesions.

Our study detected viral RNA in the liver of various wild boar with no detectable gross pathological lesions and with histological lesions almost unapparent. The histological findings associated with HEV infection pigs and wild boar have been evaluated in several studies. In experimental studies using animal models intravenously inoculated with HEV, histological findings were consistent with acute hepatitis and included ballooned hepatocytes, acidophilic bodies, focal parenchymal necrosis, severe inflammatory infiltrates in the lobules, and enlarged portal tracts [17, 19, 23], whereas in swine infected by contact, histological findings included only mild to moderate multifocal lymphoplasmacytic hepatitis and single cell necrosis of hepatocytes, which coincides with the observations in our study [17, 19, 27]. The differences in histological findings in liver between the two models of infection (intravenously inoculated and infected by contact) are probably due to the fact that in intravenous infection the diffusion of the virus in the animal is more rapid and more widespread, while in natural infection (by contact), the presence of HEV in liver may persist in the hepatic tissue over time.

Viral antigen was mainly detected in the Kupffer cells and liver sinusoidal endothelial cells, two cell populations with antigen-presenting functions residing within the sinusoidal vascular space [28, 29]. Kupffer cells are resident tissue macrophages in the liver identified by their star-shaped morphology and their location next to endothelial cells or in the endothelial lining of the liver sinusoids [30, 31]. They represent the first line of defense against viruses entering the liver through the portal vein and could play an important role in the pathogenic action of HEV, as has been observed in other liver infections [30, 32]. Further studies are required to gain a much better understanding of the mechanisms triggered by HEV to alter the functions of these cells and maintain viral infection.

The HEV RNA detected in wild boar livers in the present study (6.8%) fell within the wide range observed in some other European countries (1.9–25%), being lower than the rates of 8.3% reported in Corsica [33], 14% in south-central Italy [34], 25% in Portugal [35] and 33.5% in central Italy [36], but higher than the 1.9% of HEV-positive wild boar livers in north-western Italy [37] and 5.8% in south-western France [38]. Although no statistically significant differences were found in our study, HEV prevalence was higher in females and subadult wild boar, as previously reported. [33, 35, 37]. A similar positive correlation between young subjects and HEV infection has also been reported in domestic swine [39, 40]. Our results therefore indicate the early spread of HEV infection in young animal herds, as has been observed previously in pig facilities [41, 42].

The most important evidence of this study was the detection of HEV in the liver of naturally infected wild boar in the absence of viral RNA in serum. The immunolabeling of HEV antigen in hepatic cells confirmed infection with the virus. It has been suggested that HEV replicates in the liver of swine for only a very limited period of time [20, 23]. In humans, however, low viral titers can persist in the liver, so that viral reactivation is possible under specific conditions [43–45]. Differences between species could be related to an alteration in the mechanism of cytotoxicity responsible for limiting pathogen replication that leads to viral persistence, whereas an appropriate immune response may result in effective HEV clearance [19, 46, 47]. Given that there is a possibility of consuming contaminated raw or undercooked animal liver diagnosed as HEV-negative in serum by real-time RT-PCR, the persistence of HEV in the livers of these non-viremic wild boar is also a significant finding for public health. Several countries in different continents have reported food safety concerns associated with the detection of HEV in commercial pig liver [9, 16, 48–52], since not even heat treatment at 56°C for 1 h was able to inactivate the virus [53]. Therefore, our results give reasons to reconsider the protocols of HEV diagnosis in animals destined for human consumption and the potential risk of zoonotic transmission.

The present study assessed HEV seroprevalence in a wild boar population in the southern Spain and reported an overall seroprevalence of 5.197% (CI_95%_: 1.77–14.14). This differs from figures reported by other authors in other regions of Spain [5, 39], although our results may have been underestimated due to the low number of animals used. Curiously, the wild boar that were HEV-positive in liver showed an absence of humoral response to the virus at the time of the study. This further supports the possible theory about the lack of an adequate immune response to the virus in these animals, which coincides with other cases of persistent HEV infection seen in immunosuppressed humans, where, after HEV reactivation, no humoral response was detected for at least 6 months [43, 54].

This study also has limitations for the interpretation of results. HEV-positive liver tissue was not used as a control for RNA extraction, nor were internal/external amplification controls used to assess the possibility of false negative results, so that the prevalence of HEV in the liver could be higher. The number of animals detected with HEV infection in liver was low and the stage of infection could not be well established, since the wild boar were naturally infected.

In conclusion, this study shows evidence of HEV replication without apparent hepatitis lesions in the liver of naturally infected non-viremic wild boar. The pathogenic significance of these findings and their value for public health should be evaluated in future studies.

## Supporting information

S1 Table Histopathological evaluation of liver tissue and results of IHC, serology and real-time PCR in HEV-negative wild boar. (DOCX)
