# Supplementary material for: Persistence of hepatitis E virus in the liver of non-viremic naturally infected wild boar
Source: PLoS One. 2017 Nov 8;12(11):e0186858. doi: 10.1371/journal.pone.0186858 (PMC5678868; doi:10.1371/journal.pone.0186858)
Supplement: S1 Table — (DOCX) [file pone.0186858.s001.docx]

**S1 Table. Histopathological evaluation of liver tissue and results of IHC, serology and Real-time PCR in HEV-negative wild boar.**

| Animal | Sex | Age | Real-time PCR HEV | | IHC HEV | Ab  HEV | | Hepatocellular necrosis | | Lymphoplasmacytic  aggregates | Sinusoidal  congestion | Bile pigment accumulation |
| --- | --- | --- | --- | --- | --- | --- | --- | --- | --- | --- | --- | --- |
|  |  |  | Liver | Serum |  |  |  |  |  |  |  |  |
| 5  6  7  8  9  10  11  12  13  14  15  16  17  18  19  20  21  22  23  24  25  26  27  28  29  30  31  32  33  34  35  36  37  38  39  40  41  42  43  44  45  46  47  48  49  51  52  53  54 | Male  Female  Female  Female  Male  Male  Female  Female  Female  Female  Male  Female  Male  Male  Male  Male  Female  Female  Female  Female  Male  Female  Female  Male  Male  Male  Male  Male  Male  Female  Female  Female  Male  Female  Female  Female  Male  Female  Female  Male  Female  Female  Female  Female  Male  Female  Female  Female  Male | Adult Adult  Subadult Juvenile  Subadult  Subadult  Adult  Juvenile  Adult  Adult  Juvenile  Subadult  Adult  Adult  Adult  Adult  Subadult  Adult  Subadult  Adult  Juvenile  Juvenile  Adult  Subadult  Adult  Adult  Adult  Adult  Adult  Adult  Adult  Adult  Subadult  Adult  Adult  Subadult  Subadult  Adult  Adult  Subadult Subadult  Juvenile  Adult  Adult  Adult  Adult  Adult  Subadult  Adult | -  -  -  -  -  -  -  -  -  -  -  -  -  -  -  -  -  -  -  -  -  -  -  -  -  -  -  -  -  -  -  -  -  -  -  -  -  -  -  -  -  -  -  -  -  -  -  -  - | -  -  -  -  -  -  -  -  -  -  -  -  -  -  -  -  -  -  -  -  -  -  -  -  -  -  -  -  -  -  -  -  -  -  -  -  -  -  -  -  -  -  -  -  -  -  -  -  - | -  -  -  -  -  -  -  -  -  -  -  -  -  -  -  -  -  -  -  -  -  -  -  -  -  -  -  -  -  -  -  -  -  -  -  -  -  -  -  -  -  -  -  -  -  -  -  -  - | -  -  -  -  -  -  -  +  -  -  -  -  -  -  -  -  -  -  -  +  -  -  -  -  -  -  -  -  -  -  -  -  -  -  +  -  -  -  -  -  -  -  -  -  -  -  -  -  - | ̷  ̷  ̷  ̷  ̷  ̷  ̷  ̷  ̷  ̷  ̷  ̷  ̷  ̷  ̷  ̷  ̷  ̷  ̷  ̷  ̷  ̷  ̷  ⸹  ̷  ⸹  ̷  ̷  ̷  ̷  ̷  ̷  ̷  ̷  ̷  ⸹  ̷  ̷  ̷  ̷  ̷  ̷  ̷  ̷  ̷  ̷  ̷  ̷  ̷ | | ♦  ̷  ♦♦♦♦  ̷  ̷  ♦  ♦♦♦♦  ♦  ̷  ̷  ̷  ♦  ♦♦♦♦  ̷  ♦♦  ♦♦♦  ♦♦♦  ♦  ̷  ̷  ♦♦  ♦♦♦  ♦♦♦♦  ̷  ♦♦♦  ♦  ̷  ̷  ♦  ♦  ♦  ̷  ♦  ♦  ♦  ♦♦♦  ♦  ♦  ♦  ♦♦  ♦♦♦  ♦♦  ♦  ̷  ̷  ̷  ♦♦  ♦♦♦  ♦ | | ▲▲▲  ▲  ▲▲  ▲▲▲  ▲  ▲  ▲▲▲  ▲▲▲▲  ▲  ▲▲  ▲  ▲▲  ▲▲▲  ▲▲▲  ▲  ▲▲  ▲▲▲▲  ▲▲▲▲  ▲▲  ▲▲▲  ▲▲▲  ▲  ▲▲▲▲  ▲▲▲▲  ▲▲  ▲▲  ▲▲▲▲  ▲▲▲▲  ▲▲  ▲▲  ▲  ▲  ▲▲▲▲  ▲  ▲  ▲▲  ▲▲▲  ▲▲▲  ▲  ▲▲▲▲  ▲▲▲  ▲▲▲  ▲▲▲  ▲▲▲  ▲  ▲  ▲▲  ▲▲▲▲  ▲▲▲ | ●  ●  ●  ●●  ●  ●  ●●  ●●  ●  ●  ●  ●  ●  ̷  ●●  ̷  ●  ●  ̷  ●  ●  ̷  ●  ●  ●  ̷  ●●  ●  ●  ●●  ●  ●  ●●  ●  ●  ●  ●  ̷  ●  ●  ●  ̷  ●●  ●●  ●  ●  ●  ●  ●● |

Table S1 Legend:

Real-time PCR hepatitis E virus (HEV), immunohistochemistry (IHC) HEV and antibodies (Ab) HEV: (-) negative; (+) positive.

Hepatocellular necrosis: (̷) absence; (⸹) <10 cells per total section of 1cm^2^ approx.

Lymphoplasmacytic aggregates: (̷) absence; (♦) ≤3 small aggregates; (♦♦) 4-7 small or medium-sized aggregates; (♦♦♦) >8 small or medium-sized aggregates, or less than 4 where at least one is big; (♦♦♦♦) >8 small or medium-sized aggregates, and at least 1 big.

Sinusoidal congestion: (▲) very scarce presence of erythrocytes; (▲▲) ≈30% of sinusoids contain moderate amounts of erythrocytes; (▲▲▲) 30-80% of lobules are highly congested; (▲▲▲▲) >80% of lobules are severely congested.

Bile pigment accumulation (extracellular (canalicular cholestasis) and intracellular): (̷) absence; (●) multifocal and in low amounts; (●●) widespread and in high amounts.
